# SkelFormer: An adaptive hierarchical transformer-based approach on skeleton graphs for human action recognition in video sequences

**DOI:** 10.1371/journal.pone.0340390

**Published:** 2026-01-12

**Authors:** Jiexing Yan, Xi Zhang, Caiyan Tan, Dawen Li

**Affiliations:** 1 College of Military and Political Basic Education, National University of Defense Technology, Changsha, China; 2 School of Arts, Sun Yat-Sen University, Guangzhou, China; 3 School of Artificial Intelligence, Guangzhou University, Guangzhou, China; Bournemouth University, UNITED KINGDOM OF GREAT BRITAIN AND NORTHERN IRELAND

## Abstract

Human skeleton-based action recognition represents a pivotal field of study, capturing the intricate interplay between physical dynamics and intentional actions. Current research primarily focuses on extracting structural and temporal information from static skeleton-based graphs, but it grapples with a myriad of challenges. These include 1) an absence of hierarchical structure in encoding the skeleton-based graphs. 2) A requirement for substantial prior knowledge to interpret the diverse spatial dynamics within singular action labels. 3) An intricate task of representing the multifaceted temporal dynamics of individual actions. To address these challenges, we propose SkelFormer, a novel framework that captures spatiotemporal variations in skeleton-based graphs extracted from video sequences. The proposed SkelFormer incorporates the SKT Block as a central element, effectively facilitating information exchange through node concentration and diffusion across both structural and temporal dimensions. This design enables the extraction of hierarchical representations without relying on handcrafted rules, thereby improving the understanding of complex action patterns. Our rigorous experimental evaluations further substantiate SkelFormer’s supremacy, outperforming several state-of-the-art benchmarks in skeleton-based action recognition and achieving accuracy rates of 92.8% on the NTU RGB+D 60, 89.4% on the NTU RGB+D 120 (cross-subject split), and 96.1% on the NW-UCLA dataset.

## Introduction

Skeleton-based human action recognition is a significant advancement in computer vision, prized for its concise and powerful representation of human actions. This edge over traditional RGB data is achieved through tools like depth cameras or pose estimation algorithms. Its wide applications span surveillance systems [1,2], health monitoring [3–6], video processing [7,8], and human-computer interaction [9,10], marking its versatility in understanding intricate cognitive tasks [11].

Shifting the focus to data representation, graph neural networks (GNN) serve as a fundamental pillar in enhancing the accuracy of skeleton-based action recognition [12]. Graph-based methods have emerged as powerful tools in skeleton-based action recognition by capturing the spatial dependencies among joints and modeling temporal dynamics over sequences [13]. Instead of focusing on general graph properties, recent methods leverage the structure of human skeletal data to improve task-specific performance in action classification [14,15]. These approaches encode motion trajectories directly within the skeletal joint graph, offering more accurate recognition of fine-grained and coordinated body movements. The incorporation of temporal data into the graph structure further empowers GNN-based methodologies to effectively capture temporal dependencies across sequential frames, reinforcing their proficiency in accurately recognizing actions as they unfold over time [16]. Thus, the GNN-based approaches present a paradigm shift in the field of human action recognition, revolutionizing the interpretation of both structural interdependencies and temporal patterns inherent in human movements [17–20]

**Fig 1:**
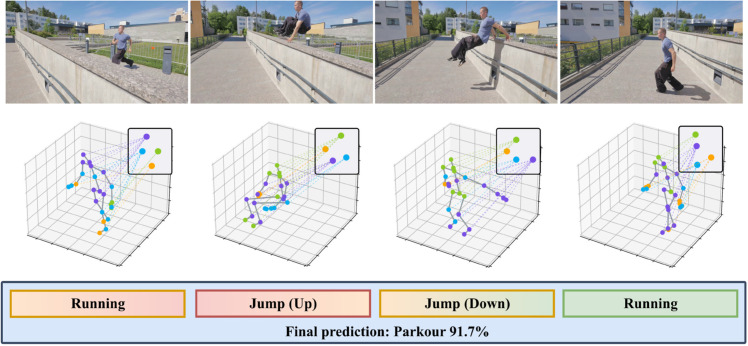
The Parkour illustration showcases four classic frames. Two capturing the spatial dynamics of “jumping” and two highlighting the temporal progression of “running.” The jumping frames contrast an energetic ascent with a controlled descent, while the running segments transition from an initial lean to synchronized movement. This highlights the complexities in graph embedding, emphasizing the challenge of capturing varied spatial and temporal nuances under a single action label.

However, the GNN-based approaches also bring three issues for future improvement. 1) The lack of a hierarchical structure presents a considerable limitation in the process of encoding a skeleton-based graph. This limitation pertains to both local graph-based structures, like individual joints and their direct spatial bonds, and the coarse-grained structure of the skeleton-based graph which represents functional units in the body. This absence of hierarchy hinders the accurate prediction of a label associated with an entire graph along the temporal dimension, obstructing a comprehensive understanding of the underlying structure of the human body and its movements [21–25]. 2) Graph embedding on the spatial structure, used to decipher dependencies between joints along temporal dimensions, necessitates the incorporation of strong prior knowledge. Indeed, the same action label, such as “jumping” as illustrated in the center of Fig 1, can represent varied spatial dynamics - the energetic upward thrust and full-body extension in jumping up differ markedly from the controlled descent and flexed landing position in jumping down, illustrating the diverse spatial and temporal nuances associated with a single action label. In Fig 1, the zoomed-in regions highlight key joint nodes and their connections across different action phases (Running, Jump (Up), and Jump (Down)). By focusing on decisive joints such as the wrists, ankles, and knees—critical to distinguishing action patterns—the figure clearly illustrates the subtle postural changes of these nodes throughout the continuous motion sequence. This visual evidence intuitively supports the design logic of SkelFormer’s window-based dynamic node composition, transforming the abstract concepts of hierarchical feature extraction and dynamic node encoding into a concrete, perceptible process. 3) The challenge in graph embedding lies in the temporal dynamics associated with a single action, which can exhibit considerable variations. Taking the example of “running” as illustrated in the left and right of Fig 1, the initial phase might depict a forward lean with arms poised for movement,

transitioning to a full stride with alternating arm swings, and culminating in a rhythmic synchronization of legs and arms propelling forward. Even though these distinct temporal graph representations are labeled under the same action, “running”, each encapsulates a unique pose or configuration.

In this work, we propose an action-driven hierarchical representation learning framework, SkelFormer, to address the challenges mentioned above. Human actions often involve multilevel coordination—from local joint movements (e.g., wrist bending) to mid-level limb patterns (e.g., arm swings), and finally to holistic postures (e.g., jumping, kicking). Capturing such structured motion dependencies requires a hierarchical representation, which is crucial for distinguishing similar actions that differ subtly in coordination levels. Without such modeling, flat representations may struggle to generalize across action contexts. Specifically, the SkelFormer is designed to capture the dynamic nature of human actions and extract hierarchical representations that capture the spatiotemporal dynamics present in the skeleton data. The pivotal component of our approach, the Skeleton Transformer(SKT) Block, is introduced to enable efficient information exchange through node concentration and diffusion modules across both structural and temporal dimensions. The node concentration module enhances the contextual understanding of each specific action by aggregating the representations of neighboring nodes in various groups on the skeleton-based graph, thereby encapsulating joint interdependencies and interactions. Subsequently, the node diffusion module extracts significant concentrated contextual representations and feeds them back to the corresponding joint along the temporal dimension.

Using the intricate movement dynamics of a parkour video as a testament to its prowess, as illustrated in Fig 1, SkelFormer masterfully encapsulates the unique topologies tied to specific actions, especially at those crucial transitional frames. The video highlights the framework’s exceptional ability to represent actions dynamically along temporal dimensions, making it ideally suited for tasks that require a nuanced understanding of human behavior. The segmentation of the “jumping” action into four temporal windows—standing, arm-swinging preparation, upward leaping, and landing—illustrates how SkelFormer automatically learns temporal transitions and segments actions accordingly. This segmentation, automatically learned through the model’s understanding of temporal dynamics, enables the system to focus on specific phases of the action, adapting to the evolving motion within the sequence. SkelFormer offers three key advantages: 1) Its unique hierarchical framework seamlessly integrates both local and coarse-grained structures, enhancing label prediction accuracy across temporal dimensions. 2) Through adaptive graph embedding, SkelFormer captures diverse spatial dynamics inherent to the same action label without heavy reliance on prior knowledge. 3) By embracing temporal variability, SkelFormer recognizes and encapsulates distinct phases within a singular action label, ensuring each unique pose is represented accurately. Achieving state-of-the-art performance, SkelFormer excels with 92.8% accuracy on the NTU RGB+D 60 4S stream and 95.0% on the NW-UCLA Joint stream. This performance is driven by its adaptive three-stage SKT architecture, which progressively abstracts features from local joint interactions to global action semantics, with relay nodes reducing from 8 to 2. This hierarchical design, combined with the node concentration and diffusion modules, allows SkelFormer to model fine-grained limb actions as well as complex full-body movements, like the dynamic transitions in “jumping.”

The main contributions in this paper are summarized as follows:

- An action-driven approach for human action recognition, SkelFormer, is designed to adeptly seize dynamic variations within skeleton-based graphs in video content. This approach extracts hierarchical representations and amplifies the comprehension of spatio-temporal dynamics, presenting a significant advancement in the field of human action recognition.
- The SKT Block dynamically captures action-specific topologies by aggregating and feeding back neighboring node representations, enhancing information exchange and contextual understanding of actions in both spatial and temporal dimensions.
- The SkelFormer framework outperforms various skeleton-based human action recognition baselines, showcasing state-of-the-art results on renowned benchmark datasets, namely NTU RGB+D 60, NTU RGB+D 120, and Northwestern-UCLA.

## Related works

### Skeleton-based action recognition

In the context of skeleton-based action recognition, graph neural networks (GNNs) have been widely adopted to model spatial and temporal relationships among human joints. Unlike traditional graph learning methods that emphasize topological properties or general graph structures, these GNN-based approaches are specifically designed to enhance the recognition of coordinated body movements across time. For example, ST-GCN [15] introduced the use of graph convolution to capture human joint connectivity and temporal motion patterns, laying the foundation for task-driven graph modeling in this domain. Despite this breakthrough, the efficiency of ST-GCN’s information propagation for high-order neighboring nodes was subpar, due to GCN’s limitations in aggregating information using adjacency matrices. To address this, AS-GCN [26] introduced the action-link, which fortified cooperative behavior features present in non-skeleton-connected joints during motion. MS-G3D [27] introduces the disentangled multi-scale aggregation scheme to effectively convey the information by reducing less significant interaction among nodes on the skeleton-based graph. While this enhancement bolstered local feature extraction, it overlooked the feature connections on long-range dependencies. ST-TR [28] attempted to solve this by employing self-attention mechanisms for structural and temporal representation extraction. Despite their efforts, they overlooked the dynamic nature of temporal information, leading to errors in predicting diverse and coherent actions. Dynamic GCN [29] utilizes CeN to automatically learn the skeleton topology, enabling a more adaptive understanding of the underlying structure and relationships within the skeleton data. However, these GCN-based approaches encounter difficulties stemming from a lack of hierarchical structure in the skeleton-based graphs, which results in inaccurate predictions of labels over temporal dimensions. Further, CTR-GCN [30] and InfoGCN [31] aims to harness contextual information through context-dependent intrinsic topology modeling and self-attention-based graph convolution, necessitating substantial prior knowledge. This introduces complexity in spatial graph embedding due to the diverse spatial and temporal nuances associated with individual action labels, thus amplifying the challenge of capturing varied temporal dynamics within a single action. In light of these complex challenges related to spatial graph embedding, hierarchical structure, and temporal dynamics, we propose SkelFormer, designed to provide a more robust and adaptive solution for human action recognition.

### Dynamic graph neural network

Incorporating temporal information into graph representations has been addressed through the utilization of Recurrent Neural Network (RNN)-based Dynamic Graph Neural Networks (DGNNs) [32,33]. These models excel at capturing temporal dynamics over time, yet their practical implementation is hindered by their high computational requirements due to the extensive graph data necessary for training. However, these approaches encounter scalability challenges when confronted with large temporal dimensions [34]. To overcome these limitations, transformer-based DGNNs [21] provide effective strategies for managing temporal information along the time dimension. For instance, TGAT [35] incorporates temporal constraints into neighborhood aggregation techniques and integrates temporal-topological representations on continuous graph datasets through a dedicated temporal graph attention layer. DySAT [36] leverages a simultaneous consideration of structural and temporal information to generate dynamic representations on discrete graph datasets. In order to convey node representation efficiently, Sparse-Dyn [37], employs a sparse temporal transformer to propagate node representations among patches and relay nodes, harnessing the power of temporal dynamics in the process.

## Methodology

### Framework overview

Recent skeleton-based human action recognition approaches face difficulties in encoding skeleton-based graphs [30,31], stemming from three key challenges: the lack of hierarchical structures hampers accurate temporal label predictions; the requirement of strong prior knowledge complicates spatial graph embedding, particularly with the diverse spatial and temporal nuances within a single action label; and the representation of varied temporal dynamics of a singular action is intricate. To address these challenges, SkelFormer has designed, an action-driven approach for human action recognition that adeptly captures dynamic variations within skeleton-based graphs in video content.

**Fig 2:**
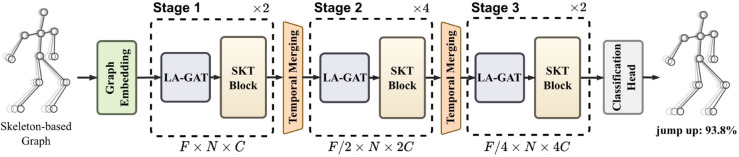
The SkelFormer’s hierarchical design consists of three stages, each anchored by an SKT Block (detailed in Fig 3). This block captures the context-sensitive topology within the spatial representation of skeleton-based graphs for specific actions. This layout allows SkelFormer to adeptly encode spatial-temporal data and adjust to the nuances of different actions.

The SkelFormer framework is designed to model the spatiotemporal dynamics of human skeleton sequences using a hierarchical architecture. As illustrated in Fig 2, the SkelFormer takes as input a skeleton-based graph representation matrix $X\in {\mathbb{R}}^{F\times N\times 3}$, where *F*, *N*, and 3 correspond to the number of frames, number of skeletal joints, and the 3D coordinates (x, y, z) of each joint, respectively. Initially, a graph embedding module applies a linear transformation to map the raw coordinates into a high-dimensional feature space, producing a representation $X\in {\mathbb{R}}^{F\times N\times C}$, with *C* = 64 by default. This transformation encodes the low-level joint features for downstream spatiotemporal modeling.

To capture hierarchical semantics, SkelFormer introduces a three-stage processing pipeline. In stage *i*, the feature dimension of *X* is scaled to *C* × 2^(*i*−1)^, thereby increasing representational capacity as the network deepens. Each stage consists of two core components: a Learnable-Adjacency Graph Attention (LA-GAT) module that adaptively learns spatial relationships between joints, and an SKT Block that extracts dynamic spatiotemporal patterns. Between adjacent stages, a temporal merging operation is applied via a 1D convolutional layer with both stride and kernel size set to 2, effectively downsampling the temporal dimension from *F* to *F*/2^(*i*−1)^. This progressive abstraction through spatial and temporal hierarchies enables SkelFormer to robustly model complex action semantics. Finally, the output representation is passed to a classification head, which predicts the action label.

### Learnable-Adjacent GAT

In addressing the rapid attenuation of information across extended distances on graphs with fixed adjacency matrices, the innovative approach of LA-GAT is introduced. Drawing inspiration from InfoGCN [31], LA-GAT incorporates learnable parameters into its architecture, optimizing node information transfer. This integration is enriched through ablation studies that assessed various initialization methodologies, ultimately leaning towards initializing with the original adjacency matrix to bolster performance. By leveraging the robust capabilities of gradient computation, LA-GAT achieves a nuanced understanding of skeletal structures. Furthermore, it enhances the efficient distribution of node information. This concept is mathematically depicted in Eq 1.

$$SA_{ij}={\mathrm{Softmax}}_{j}\left(\alpha \left(Wx_{i},Wx_{j}\right)\right)⊙LA_{ij}$$ (1)

Where *SA*_*ij*_ depicts the importance of node *j*’s features to node *i*, $X=\left\{x_{1},x_{2},...,x_{N}\right\}$ is a collection of joint features. The shared linear transformation applied to each node is represented by $W\in {\mathbb{R}}^{C\times C}$. The term α signifies the computation of the attention coefficient, the ⊙ stands for the Hadamard product, whereas *LA* stands for the learnable parameter initialized using the adjacency matrix, and *LA*_*ij*_ depicts the connection strength of node *j*’s features to node *i*. This carefully crafted equation, along with its parameters, underscores the versatility and precision of LA-GAT in capturing intricate graph-based relationships.

Building upon this formulation, LA-GAT draws inspiration from models such as GAT [38] and InfoGCN by embedding attention mechanisms into its computational framework. However, during the spatio-temporal attention computation, we observed that projecting *X* into the full QKV space introduced redundancy, which negatively impacted model accuracy. To mitigate this, LA-GAT streamlines the process by projecting *X* only into the QK space, while leveraging the original feature matrix *X* for value aggregation, guided by the attention coefficients defined in the preceding equation. Notably, the projection of *X* into the *V* space is delegated to the subsequent SKT Block, ensuring modularity and reducing unnecessary computation.

### SKT block

**Fig 3:**
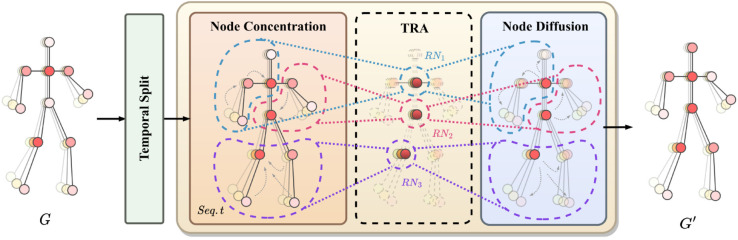
Structure of SKT Block. The illustrated block details the progression of action-driven representations on dynamic temporal skeleton-based graphs, incorporating three key operations: temporal split, node concentration, and node diffusion. This arrangement enables a switch between global and concentrated representations, thereby enhancing the interpretation of the processed behavioral information. With the integration of these operations, the model efficiently encapsulates the dynamic aspects of the actions, bolstering the overall process of representation learning.

By leveraging the hierarchical structure of skeletal graphs, the SKT Block—a core component of the proposed framework—is developed to model the dynamic evolution of human poses over time, enabling the capture of subtle transitions in skeletal actions. As illustrated in Fig 3, the SKT Block consists of three functional submodules. The Temporal Split submodule divides long action sequences into localized temporal segments, enabling the model to focus on meaningful motion phases with multi-scale temporal resolution. The Node Concentration submodule dynamically groups spatially related joints into semantic clusters, enabling the abstraction of functional motion units, such as limbs or coordinated body parts. Beyond simple aggregation, this process emphasizes anchor nodes with consistent temporal importance, enabling the model to capture subtle yet discriminative variations in joint dynamics (e.g., the nuanced differences in wrist or ankle movements across phases of an action). Subsequently, the node diffusion submodule redistributes these aggregated contextual features back to individual joints along the temporal dimension. This bidirectional exchange of information—concentration from local joints to abstract clusters, and diffusion from clusters to individual joints—ensures that fine-grained local dynamics are aligned with global semantic understanding. Such a mechanism enables the SKT Block to not only construct hierarchical spatiotemporal representations but also to explicitly model the flow of information that underlies the decision-making process in action recognition. A comprehensive exploration of the functionalities and intricacies of each submodule will be detailed in the subsequent sections.

#### Temporal split.

In recent human action recognition studies, multi-scale temporal convolution has become a staple for the extraction of multi-scale temporal data [30,31]. By leveraging convolutional branches distinguished by varying kernel sizes and dilation rates, it affords precise encoding across multiple temporal scales [27]. However, a significant limitation underpins this approach: it neglects the integration of the graph’s hierarchical information. Consequently, while extracting temporal nuances, the system overlooks spatial interrelations with other potential nodes in the graph’s hierarchy.

We present the novel Temporal Split module, designed to bridge existing gaps and synergize with the SKT Block. It enables the concurrent assimilation of hierarchical data through the Transformer mechanism by segmenting the input time series into non-overlapping *F*/*T* sections, each containing *T* frames. Within these segments, the SKT Block performs self-attention, offering insights into temporal dynamics. Our design distinctively incorporates Temporal Merging. When applied across stages, it reduces the time series length by half, endowing the model with an expansive temporal view. However, there’s a risk of attention fragmentation and loss of temporal connections. To mitigate this, we alternate segmentation strategies across successive SKT Block layers, interweaving original time series sequences with those offset by half a segment, thereby ensuring sustained attention and enhancing the model’s expressiveness.

The motivation behind this design stems from the observation that human actions often unfold in temporally discrete stages—for instance, “prepare”, “jump”, and “land” in a jumping sequence. Each stage may engage different joint groups or motion primitives. By aligning temporal segmentation with the model’s hierarchical spatial reasoning, the Temporal Split module enables the network to better capture these stage-wise transitions and associate them with corresponding functional body structures. This joint spatiotemporal structuring is crucial for accurately recognizing complex and dynamic human actions.

#### Nodes concentration module.

The node concentration module’s capacity to capture fine-grained dynamic changes stems from its multi-stage process of relational reasoning and feature distillation. Rather than treating all temporal fluctuations as a signal, the module intelligently discriminates between salient motion patterns and irrelevant noise by leveraging both spatial and temporal contexts. The mechanism is not a simple aggregation but a refined clustering that preserves critical local dynamics within a structured hierarchy.

The process initiates with the computation of the spatial attention matrix *SA*, whose elements *SA*_*ij*_ represent the directed influence from node *i* to node *j*. To infuse this spatial affinity with temporal awareness, we compute a time-conditioned importance score for each node. Let $H^{\left(t\right)}=[h_{1}^{\left(t\right)},h_{2}^{\left(t\right)},...,h_{N}^{\left(t\right)}{]}^{T}$ be the node features at time *t*. The spatial attention coefficient at a specific frame is calculated in Eq 2.

$$SA_{ij}^{\left(t\right)}=\frac{\exp\left(\sigma \left({𝐚}^{T}[𝐖{𝐡}_{i}^{\left(t\right)}\|𝐖{𝐡}_{j}^{\left(t\right)}]\right)\right)}{\sum_{k\in {𝒩}_{i}}\exp\left(\sigma \left({𝐚}^{T}[𝐖{𝐡}_{i}^{\left(t\right)}\|𝐖{𝐡}_{k}^{\left(t\right)}]\right)\right)}$$ (2)

where **W** is a shared weight matrix, **a** is a weight vector, σ is the LeakyReLU activation, and ‖ denotes concatenation. The subsequent temporal pooling, $\sum_{t=1}^{T}{\mathrm{AvgPooling}}_{t}(SA)$, is therefore not an average over static relations but an integral of dynamically evolving interactions. A node accrues a high importance score only if it consistently participates in strong spatial affiliations throughout the action sequence. This ensures that the selected Anchor Nodes, *X*_*AN*_, are hubs of persistent activity, which is critical for characterizing the essence of the action.

The core of fine-grained capture lies in the group formation and feature concentration. The similarity metric $\mathrm{Sim}(X,X_{AN})$ is calculated in Eq 3.

$$S=\mathrm{Softmax}\left(\frac{\phi (X)\psi (X_{AN}{)}^{T}}{\sqrt{d}}\right)$$ (3)

where ϕ(·) and ψ(·) are independent non-linear projections (e.g., single-layer MLPs). The use of separate projection functions enables the model to learn a specialized similarity space where nodes are grouped based on functional motion roles rather than mere spatial proximity. The subsequent HardMax operation, Group=HardMax(S), creates a strict, disjoint partition $Group\in {\mathbb{R}}^{N\times K}$. This forces the model to make a definitive assignment, sharpening the semantic boundaries between different functional units (e.g., a hand node is decisively grouped with the arm anchor rather than ambiguously shared with a torso anchor).

The concentration of features into relay nodes *X*_*RN*_ is where local dynamics are distilled. The operation $X_{RN}=\Lambda^{-1}Group^{T}X$ is a weighted aggregation. We can deconstruct this to understand its effect on dynamic information. The feature of a relay node *k* is calculated in Eq 4.

$${𝐱}_{RN}^{\left(k\right)}=\frac{1}{\left|{𝒢}_{k}\right|}\sum_{i\in {𝒢}_{k}}{𝐱}_{i}$$ (4)

where ${𝒢}_{k}$ is the set of nodes assigned to anchor *k*. This averaging acts as a low-pass filter, suppressing high-frequency noise (e.g., sensor jitter in a single joint) while reinforcing the common motion signal shared by all nodes in the group. If all joints in a “hand” group rotate in concert during a wave gesture, their shared trajectory is amplified in ${𝐱}_{RN}^{\left(k\right)}$, while the independent jitter of a single joint is averaged out. Consequently, the relay node representation *X*_*RN*_ encapsulates a purified and enhanced signal of the coordinated, fine-grained movements that define the action, providing a robust and hierarchical basis for all subsequent processing stages. The implementation of the node concentration module is as Algorithm 1.

**Algorithm 1 Hierarchical node concentration.**

**Input**: Spatial Attention Matrix $SA_{N\times N}$, Node Features $X_{N\times C}$, Number of Anchors *K*

**Output**: Relay Nodes Feature $X_{RN_{K\times C}}$

1: **Step 1: Anchor Node Selection via Temporal Persistence**

2: Compute temporal importance scores: $I=\sum_{t=1}^{T}{\mathrm{AvgPooling}}_{t}(SA)$

3: Select anchor indices: 𝒜=TopK−Indices(I,K)

4: Extract anchor features: $X_{AN}=X[𝒜,:]$

5: **Step 2: Semantic Group Formation**

6: Project features: $\phi (X)=\mathrm{GeLU}(XW_{\phi })$, $\psi (X_{AN})=$ $\mathrm{GeLU}(X_{AN}W_{\psi })$

7: Compute similarity matrix: $S=\mathrm{Softmax}\left(\frac{\phi (X)\psi (X_{AN}){\;}^{T}}{\sqrt{d}}\right)$

8: Hard assignment: G=HardMax(S) *G*_*ij*_ = 1 if node *i* belongs to group *j*

9: **Step 3: Feature Concentration with Dynamic Normalization**

10: Initialize relay nodes: $X_{RN}=G^{T}X$ Accumulate features per group

11: Compute group cardinalities: $\Lambda =\mathrm{diag}(G^{T}1_{N})$

12: Normalize: $X_{RN}=\Lambda^{-1}X_{RN}$ Average pooling within clusters

13: **return** *X*_*RN*_

SkelFormer’s dynamic approach to node concentration marks a significant advancement in capturing the fine-grained temporal dynamics of human actions. Unlike traditional skeletal representations that rely on fixed graph topologies, SkelFormer adaptively forms semantically meaningful clusters of nodes based on learned attention relevance. This allows the model to evolve its groupings over time, ensuring that the focus is on task-relevant joint interactions rather than rigid, anatomically predefined joint relationships. As a result, the model is capable of capturing subtle temporal variations in human actions, which is crucial for recognizing complex actions that involve nuanced, isolated movements of individual joints. Furthermore, the use of node diffusion in the later stages propagates the aggregated semantic representations back to individual joints, ensuring context-aware refinement of node features across time. This dynamic and flexible design enables SkelFormer to break free from the limitations of fixed joint positions, aligning more closely with the functional motion patterns inherent in human action dynamics.

#### Nodes diffusion module.

The Node Diffusion (ND) module constitutes the complementary top-down pathway in the SkelFormer architecture, designed to propagate globally refined temporal context back to the local node-level representations. Following the abstraction performed by the Node Concentration module, which yields a set of semantically coherent relay nodes $X_{RN}\in {\mathbb{R}}^{K\times T\times C}$, the ND module is tasked with temporal reasoning and the subsequent dissemination of this information. Its primary function is to ensure that the nuanced, fine-grained dynamics of individual joints are not lost in the hierarchical abstraction but are instead enriched by a global temporal perspective, thereby creating a cohesive spatiotemporal representation.

The core of this temporal reasoning is a novel Temporal Relay Attention (TRA) mechanism. Unlike standard self-attention that operates on a flat node structure, TRA computes dependencies among the *K* relay nodes across the temporal dimension. The Query and Key vectors are derived explicitly in Eq 5 from the relay nodes themselves through independent linear projections.

$$Q=X_{RN}W_{Q},\;K=X_{RN}W_{K}$$ (5)

where $W_{Q},W_{K}\in {\mathbb{R}}^{C\times d_{k}}$ are learnable projection matrices. This formulation allows the model to learn interactions between functional body units over time by evaluating the compatibility between the query of one relay node and the key of another. The attention coefficients are then computed in Eq 6.

$$\text{TRA}=\mathrm{Softmax}\left(\frac{QK^{⊤}}{\sqrt{d_{k}}}\right)$$ (6)

The resulting tensor $\text{TRA}\in {\mathbb{R}}^{K\times T\times T}$ encodes the influence of relay node *j* at time $t^{\prime }$ on relay node *i* at time *t*. A critical design choice is the decoupling of the Value source. The Value matrix is not projected from *X*_*RN*_, but from the original node features $X\in {\mathbb{R}}^{N\times T\times C}$ in Eq 7.

$$V=XW_{V}$$ (7)

where $W_{V}\in {\mathbb{R}}^{C\times d_{v}}$. This ensures that the information being aggregated and redistributed is anchored in the fine-grained, high-dimensional features of the original joint set, preserving the potential for reconstructing local dynamic details.

The actual diffusion, or top-down information flow, is executed through a tensor contraction that leverages the grouping matrix *G* from the concentration phase. The updated temporal context is distributed to all nodes as follows in Eq 8

$$X^{\prime }=G\cdot \text{TRA}\cdot V$$ (8)

where, $G\in {\mathbb{R}}^{N\times K}$ is the one-hot group assignment matrix. This operation can be deconstructed into two semantically meaningful steps. First, the TRA-weighted Value matrix, TRA · V, performs a temporal aggregation, producing a contextually enriched set of relay node features ${\tilde{X}}_{RN}\in {\mathbb{R}}^{K\times T\times d_{v}}$. Subsequently, the multiplication with the group matrix *G* performs a broadcast operation in Eq 9.

$$x_{i,t}^{\prime }=\sum_{k=1}^{K}G_{i,k}\cdot {\tilde{x}}_{RN}^{\left(k,t\right)}$$ (9)

This assigns the updated feature vector of relay node *k* at time *t* to every original node *i* that belongs to group *k*. Consequently, all joints within a functional group (e.g., the entire arm) receive the same temporally refined contextual information. This process captures fine-grained dynamics by ensuring that the localized motion of a joint (e.g., a subtle wrist flexion) is now interpreted within the broader context of its functional unit’s behavior across the entire action sequence. The final output $X^{\prime }$ is a node-level representation where each joint’s features are dynamically aligned with the global spatiotemporal evolution of the action, significantly enhancing the model’s discriminative power for complex activity recognition.

### Action prediction

Following the hierarchical spatiotemporal processing through the SKT Blocks, the model generates a rich, high-dimensional feature tensor. To transition from this structured representation to a definitive action classification, a robust prediction and optimization pipeline is employed. The refined feature map, possessing dimensions [Batch,T,N,C], is first condensed into a singular, global descriptor vector per sequence. This is achieved via a global averaging pool, which aggregates features across the temporal, spatial, and embedding dimensions. Specifically, the operation is computed in Eq 10.

$${𝐳}_{n}=\frac{1}{T\cdot N\cdot C}\sum_{t=1}^{T}\sum_{i=1}^{N}\sum_{c=1}^{C}𝐗[n,t,i,c]$$ (10)

where ${𝐳}_{n}\in {\mathbb{R}}^{C}$ is the aggregated feature vector for the *n*-th sample in the batch. This global averaging operation serves as a strong inductive bias, enforcing an equal weighting of all spatiotemporal contexts and producing a compact, holistic representation of the entire action sequence. The resulting vector ${𝐳}_{n}$ is then passed through a linear classifier, parameterized by a weight matrix $W_{cls}\in {\mathbb{R}}^{C\times D}$ and a bias term *b*, to generate the final logits for each action class: ${\hat{y}}_{n}=W_{cls}z_{n}+𝐛$.

To ensure robust generalization and mitigate overfitting, which is a common challenge in deep models with high-dimensional skeletal data, the training objective utilizes Label Smoothing Cross-Entropy [39] (LSCE) in Eq 11.

$${ℒ}_{\text{LSCE}}=-\frac{1}{N}\sum_{n=1}^{N}\left[\alpha \sum_{c=1}^{C}y_{n,c}\log({\hat{y}}_{n,c})+(1-\alpha )\sum_{c=1}^{C}\left(\frac{1}{C}\right)\log({\hat{y}}_{n,c})\right]$$ (11)

where the smoothed label distribution *q*_*c*_ is explicitly defined as a uniform distribution $\frac{1}{C}$. The smoothing factor α calibrates the model’s confidence penalty. This mechanism prevents the model from becoming overconfident by discouraging the maximization of the logit for the ground-truth class to the exclusion of all others. By incorporating a prior that all classes are somewhat plausible, LSCE acts as an effective regularizer, improving calibration and enhancing the model’s ability to discriminate between subtle, fine-grained action classes on unseen data. This combination of a globally pooled feature representation and a confidence-penalizing loss function ensures that SkelFormer delivers accurate and well-generalized action predictions.

## Results

### Experiments setting

SkelFormer, built on PyTorch [40], is trained on an NVIDIA RTX 3090 GPU and uses a warm-up cosine scheduler. On the NTU RGB+D series dataset [41], we set the batch size to 128, the learning rate to 0.1, and weight decay to 0.0005. We trained for 110 epochs, including a 5-epoch warm-up, using a random seed of 2023. On the Northwestern-UCLA dataset [42], we configured a batch size of 8, a learning rate of 0.01, and a weight decay of 0.0005. The training spanned 60 epochs, inclusive of a 5-epoch warm-up, and utilized a random seed of 2023. The relay nodes’ counts in their three stages are 8, 4, and 2. For the NTU dataset, it uses a frame sequence length (*F*) of 64 and a Temporal Split length (*T*) of 16. For the UCLA dataset, these are set to 52 and 13, respectively.

### Datasets

**NTU RGB+D 60** [41]. The NTU RGB+D 60 dataset stands as a benchmark in skeleton-based human action recognition, boasting 56,880 skeletal action sequences. These sequences are derived from 40 subjects and are categorized into daily actions, health-related activities, and mutual actions. Captured using the Microsoft Kinect-V2 depth sensors, the actions are recorded under 17 distinct scene conditions, offering a rich variety of data. Additionally, this dataset provides a comprehensive set of multi-modal information, including depth maps, 3D skeleton joint positions, RGB frames, and infrared sequences. Evaluation criteria for this dataset emphasize both Cross-Subject (X-Sub) and Cross-View (X-View) standards, reflecting its comprehensive and versatile nature.

**NTU RGB+D 120** [41]. The NTU RGB+D 120 dataset encompasses a broader range of human actions with more than 114 thousand video samples and over 8 million frames. This extensive dataset, collected from 106 unique subjects, encapsulates 120 diverse action classes, spanning daily, mutual, and health-related activities. Serving as a more extensive reference for RGB+D human action recognition, the NTU RGB+D 120 also introduces an additional evaluation standard, the Cross-Setup (X-Setup), alongside the conventional Cross-Subject (X-Sub) standard. This augmentation in both content and evaluation criteria underscores the dataset’s significance and applicability in the realm of human action recognition.

**Northwestern-UCLA** [42]. The Northwestern-UCLA dataset is a comprehensive collection of 1494 video sequences, meticulously annotated with 20 joint-based skeletons to provide granular spatial information for advanced analysis. These sequences encompass 10 distinct action categories, namely: pick up (using one or two hands), drop trash, walk around, sit down, stand up, donning, doffing, throw, and carry. Each of these actions has been performed by 10 different actors, ensuring diversity in the dataset. Captured using three synchronized Kinect cameras, the dataset boasts RGB, depth, and human skeleton data, offering a multi-faceted perspective by including data from a variety of viewpoints. This rich diversity makes the Northwestern-UCLA dataset a valuable resource for in-depth human action analysis.

### Evaluation of human action recognition

Table 1 demonstrates that SkelFormer achieves consistently strong performance on the NTU RGB+D 60 and 126 datasets across both the X-Sub and X-View evaluation protocols. By leveraging multiple input modalities—including the Joint (J), Bone (B), and Four-Stream (4S) configurations—SkelFormer surpasses existing baselines. Specifically, when benchmarked against InfoGCN [47], it achieves notable accuracy improvements: +0.8%, +1.0%, and +0.1% on the X-Sub subset using the J, B, and 4S streams respectively, and +0.3% and +0.2% on the X-View subset using the J and B streams on the NTU RGB+D 60 dataset. These consistent gains across different data streams underscore the effectiveness of SkelFormer’s hierarchical representation learning, which enhances its ability to capture subtle spatiotemporal patterns critical for discriminating between complex human actions.

**Table 1:** Quantitative comparison demonstrating the performance of different approaches on the NTU RGB+D 60 and NTU RGB+D 120 datasets. Four metrics, J, B, and 4S, are evaluated, with the highest accuracy scores highlighted in bold font. It should be noted that for some methods, certain indicators cannot be computed and are thus represented by ‘-’.

Setting	NTU 60								NTU 120							
	X-Sub				X-View				X-Sub				X-Set			
Method/Modality	J	B	J+B	4S	J	B	J+B	4S	J	B	J+B	4S	J	B	J+B	4S
ST-TR [28]	89.2	-	90.3	-	95.8	-	96.3	-	82.7	-	85.1	-	85.0	-	87.1	-
Shift-GCN [43]	87.8	-	89.7	90.7	95.1	-	96.0	96.5	80.9	-	85.3	85.9	83.2	-	86.6	87.6
Dynamic GCN [29]	-	-	-	91.5	-	-	-	96.0	-	-	-	87.3	-	-	-	88.6
MS-G3D [27]	89.4	90.1	91.5	-	95.0	95.3	96.2	-	-	-	86.9	-	-	-	88.4	-
MST-GCN [44]	89.0	89.5	91.1	91.5	95.1	95.2	96.4	96.6	82.8	84.8	87.0	87.5	84.5	86.3	88.3	88.8
2S-AGCN [45]	88.9	89.2	91.0	91.5	94.5	94.1	95.7	95.9	84.0	85.1	87.8	88.2	85.3	86.3	89.0	89.6
CTR-GCN [30]	89.8	90.2	92.0	92.4	94.8	94.8	**96.3**	**96.8**	84.9	85.7	**88.7**	88.9	86.7	87.5	90.1	90.5
InfoGCN^*^ [31]	89.4	90.6	91.3	92.3	95.2	95.4	96.2	96.7	84.2	86.9	88.2	89.2	86.3	88.5	89.4	90.7
SkelFormer	**90.6**	**91.6**	**92.0**	**92.8**	**95.5**	**95.7**	96.2	96.7	**86.1**	**87.8**	88.6	**89.4**	**87.4**	**89.3**	**90.1**	**91.0**

SkelFormer’s excelling becomes even more pronounced on the more challenging NTU RGB+D 120 dataset. On the X-Sub subset, it achieves accuracy gains of +1.0% and +1.5% over InfoGCN for the Joint and Bone streams, respectively (InfoGCN using extra MMD losses, and the result in Table 2 comes from retraining the models using InfoGCN’s officially released code.). Similarly, on the X-Set subset, SkelFormer maintains its lead with improvements of +1.1% (J) and +0.8% (B). These results highlight the model’s robustness and generalizability across a broader range of action classes and intra-subject variations. Crucially, SkelFormer’s hierarchical modeling enables it to better distinguish between actions with highly similar joint trajectories by encoding both local and global relational cues. This structural expressiveness proves indispensable for tackling the fine-grained motion differences characteristic of the NTU RGB+D 120 dataset.

**Table 2:** Quantitative comparison demonstrating the performance of different approaches on the NW-UCLA datasets. The metrics and indicators of the record in this table are labeled the same as in Table 1.

Dataset	NW-UCLA		
Method/Modality	J	B	4S
AGC-LSTM [46]	93.3	-	
Shift-GCN	92.5	-	94.6
2S-AGCN	92.0	92.2	95.5
CTR-GCN	94.6	91.8	**96.5**
InfoGCN	93.8	94.2	96.1
SkelFormer	**95.0**	**94.2**	96.1

Table 2 demonstrates that SkelFormer maintains its competitive advantage on the NW-UCLA dataset, achieving state-of-the-art performance in both the Joint and Bone streams with accuracies of 95.0% and 94.2%, respectively. Although it ranks second in the Four-Stream configuration, it trails the leading model CTR-GCN by only 0.4%, thereby reaffirming its high efficacy even in compact and diverse action sets. These results can be attributed to SkelFormer’s explicit encoding of hierarchical graph information, which allows the model to capture both coarse and fine motion dynamics adaptively. The visualization section further confirms that SkelFormer can effectively isolate subtle transitions and overlapping trajectories, which are often sources of confusion in skeleton-based recognition. Beyond accuracy, SkelFormer also delivers reduced computational overhead and swift inference, making it not only powerful but also practical for real-world applications.

### Ablation study

#### Initialization of LA-GAT.

Table 3 a) presents the impact of three distinct initialization techniques for LA-GAT. While there’s a negligible difference in SkelFormer’s performance between random initialization and identity matrix initialization, leveraging original adjacency information as an initialization method enhances SkelFormer’s performance by 0.3% on the NTU RGB+D 60 dataset. This improvement is likely attributed to the inherent advantages of utilizing the prior knowledge from the original adjacency details of the skeleton-based graph.

**Table 3:** Ablation Studies of Bone Stream on NTU RGB+D 60 dataset. The modules definition: B: Baseline; TS: Temporal Split; SC: Static Concentration; ND: Node Diffusion; DC: Dynamic Concentration. The highest accuracy score is denoted in bold font, while the green color indicates an accuracy improvement when compared to the baseline.

(a) **Initialization**. The table shows the effect of different LA parameter initialization methods on the model accuracy of LA-GAT. We experiment with random, identity matrix, and adjacency matrix three initialization methods.	
**Adapter matrix Init**	**Acc(%)**
Random	91.3
Identity matrix	91.3
Adjacent matrix	**91.6** +0.3
(b) **Performance Impact of Incrementally Integrating SKT Sub-Modules.** The baseline model substitutes the entire SKT block with a conventional transformer architecture while maintaining an identical experimental setup.	
**Block Design**	**Acc(%)**
Baseline	90.3
B + TS	90.7 +0.4
B + SC + ND	90.6 +0.3
B + TS + SC + ND	90.9 +0.6
B + TS + DC + ND	**91.6** +1.3

#### Impact of SKT block components.

Table 3 deconstructs the SKT block to quantify the contribution of its core modules, beginning with a baseline model (B). This baseline substitutes the SKT block with a conventional temporal attention mechanism applied to the complete node sequence, establishing a performance reference point. The incremental integration of components reveals their distinct roles: introducing the Temporal Split (TS) module, which processes the sequence through a sliding-window mechanism over local temporal segments, yields a modest accuracy improvement of +0.4% over the baseline. This confirms the initial hypothesis that localized temporal modeling is more effective than global sequence attention in capturing action dynamics. Subsequently, integrating Static Concentration (SC)—which imposes a fixed, anatomical prior by grouping nodes into five predefined clusters (e.g., arms: [9,10,11,12,24,25], legs: [17,18,19,20]—provides a further marginal gain of +0.3%. This suggests that explicit structural coherence aids feature extraction, even when the grouping is not adaptive.

The synergistic effect of combining these modules with the Node Diffusion (ND) mechanism is clearly demonstrated. The integration of TS, SC, and ND produces a combined gain of +0.6% over the baseline. This highlights that the diffusion of relational information back to individual nodes is not merely additive but essential for complementing the local temporal context provided by TS and the structural prior enforced by SC. However, the most pivotal advancement is realized by transitioning from static to dynamic node concentration (DC). Unlike the fixed partitioning of SC, dynamic concentration computes node clusters adaptively, conditioned on the input sequence according to the attention-based grouping mechanism defined by $Group=\mathrm{HardMax}(\mathrm{Sim}(X,X_{AN}))$. This enables the model to capture fine-grained, action-specific variations in joint correlations that are not present in a rigid anatomical prior.

When the full suite of dynamic components—TS, DC, and ND—is assembled, the model achieves a significant accuracy of 91.6%, representing a +1.3% gain relative to the baseline. This superior performance underscores that the principal strength of the SKT block lies not merely in leveraging temporal locality and structural priors in isolation, but in its capacity to dynamically regulate the flow of information. The mechanism allows the model to selectively amplify features from discriminative joints while attenuating less informative ones through a learned, input-dependent hierarchy. The findings conclusively demonstrate that while incremental gains can be achieved through static priors, the dynamic methodology embodied by the whole SKT block, characterized by its adaptive concentration and diffusion processes, is indispensable for achieving robust and state-of-the-art action recognition.

### Visualization

#### Action-driven node concentration.

**Fig 4:**
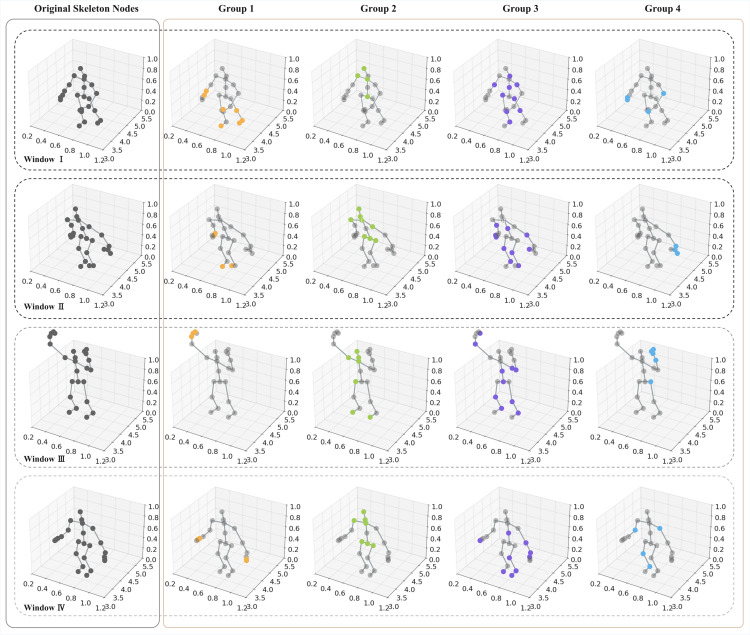
A graphical depiction of SkelFormer’s action-driven hierarchical embedding learning. The left gray box represents the temporal sequence of a given action, while its spatial node aggregations for each phase on the skeleton-based graph are highlighted in the right brown box.

Fig 4 illustrates SkelFormer’s capability to learn node groupings in action-driven scenarios by segmenting the “jump” action into four temporal windows: standing, arm-swinging preparation, upward leaping, and landing. This progression highlights how node concentration dynamically evolves across temporal dimensions, reflecting the underlying structural changes of the action. While the figure primarily depicts the outcomes of Stage 3, where only two relay nodes are retained to abstract global semantics, the trends observed are consistent with the hierarchical design of SkelFormer. Specifically, Stage 1 emphasizes fine-grained joint interactions using eight relay nodes to capture localized dependencies, such as elbow–wrist coordination, while Stage 2 employs four relay nodes to integrate larger regional dependencies like arm–trunk coupling. Thus, although the first two stages are not explicitly visualized in the figure, the observed temporal progression of node groupings from windows 1–4 echoes the model’s staged transition from local to global representations.

In addition, Fig 4 highlights the evolving spatial dynamics of node positions on the skeleton graph, demonstrating how nodes adaptively reconfigure into action-dependent groups. Nodes with high levels of feature aggregation are distinctly color-coded, revealing that during different phases of the action, key joints dynamically merge to form unique clusters. The number and composition of these clusters change across temporal windows, particularly between static postures such as standing and dynamic movements like leaping. This adaptability underscores the strength of SkelFormer’s node diffusion and concentration mechanisms, which enable the model to capture fine-grained dynamic changes and progressively integrate them into robust global action semantics.

#### Performance on subtle limb actions.

**Fig 5:**
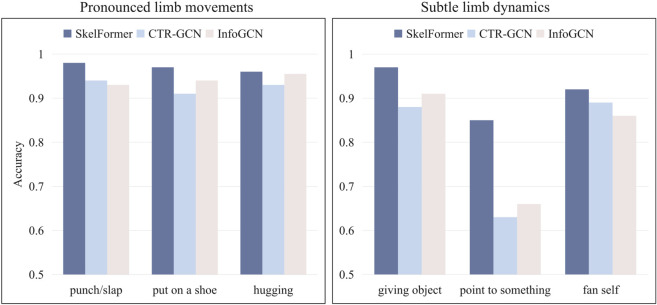
Comparative visualization of action recognition approaches. The left panel focuses on actions with distinct limb movements, while the right panel showcases those with more subtle limb dynamics.

Fig 5 illustrates a comparative analysis of action recognition accuracy among SkelFormer, CTR-GCN, and InfoGCN on the NTU RGB+D 60 Bone stream, highlighting key differences in model performance based on action complexity. For actions involving pronounced limb and torso movements—such as “punch/slap,” “put on a shoe,” and “hugging”—all models achieve good accuracy, suggesting that contemporary skeleton-based approaches are highly effective for well-defined, full-body motions. However, a more nuanced picture emerges when examining subtle actions dominated by fine-grained joint interactions (e.g., “pointing to something,” “giving an object,” and “fanning oneself”). Here, CTR-GCN’s performance deteriorates significantly (63% for “pointing”), while InfoGCN plateaus below 70%, indicating that conventional graph-based methods struggle to capture localized motion patterns. In contrast, SkelFormer demonstrates remarkable robustness ( 85% accuracy), likely due to its hierarchical attention mechanism, which enables multi-scale feature learning—effectively bridging global pose dynamics with local joint relationships. This performance gap highlights a critical limitation in existing GCN-based approaches: their reliance on predefined bone connectivity may hinder their adaptability to actions that require precise, context-dependent joint correlations. SkelFormer’s superior performance on such challenging cases suggests that transformer-based architectures, with their self-attention mechanisms, offer a more flexible framework for modeling intricate skeletal dependencies. Future work could explore hybrid architectures that combine the strengths of GCNs and transformers to further improve generalization across diverse action types.

**Fig 6:**
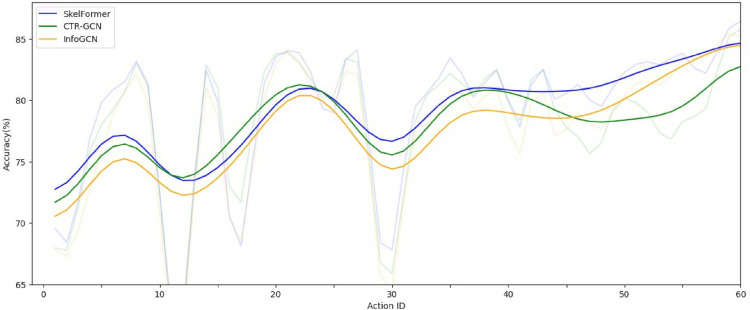
Visual comparison of human action predictions of SkelFormer with InfoGCN and CTR-GCN in NTU RGB+D 60 dataset Bone Stream. The accuracy curve is smoothed to observe the stability of the model prediction.

Fig 6 illustrates a comparative analysis of human action prediction performance among SkelFormer, CTR-GCN, and InfoGCN on the NTU RGB+D 60 dataset’s Bone Stream, where model outputs are processed using the softmax mechanism [48]. The visualization presents both raw accuracy (depicted as semi-transparent shading) and a smoothed trajectory (solid line) to better assess model stability across all 60 action categories. Notably, SkelFormer demonstrates superior performance in 80% of cases (48 out of 60 actions), with its smoothed accuracy curve exhibiting significantly less volatility compared to competing models. This enhanced stability is particularly evident in actions involving fine motor control or subtle joint movements, where traditional GCN-based approaches, such as CTR-GCN and InfoGCN, exhibit pronounced performance fluctuations. The consistency of SkelFormer’s predictions suggests its transformer-based architecture may better capture long-range dependencies and temporal dynamics in skeletal data compared to graph convolution methods. Furthermore, the performance gap widens in categories requiring nuanced interpretation of joint relationships, highlighting a fundamental advantage of attention mechanisms in processing hierarchical skeletal features. These findings have important implications for real-world applications where prediction stability is crucial, such as healthcare monitoring or human-robot interaction systems. Future research directions might explore the integration of SkelFormer’s attention mechanisms with spatio-temporal graph representations to further enhance performance on challenging action categories while maintaining computational efficiency. The results also prompt reconsideration of traditional evaluation metrics, suggesting that stability measures should complement accuracy in assessing model robustness for deployment in variable real-world conditions.

## Discussion

**Fig 7:**
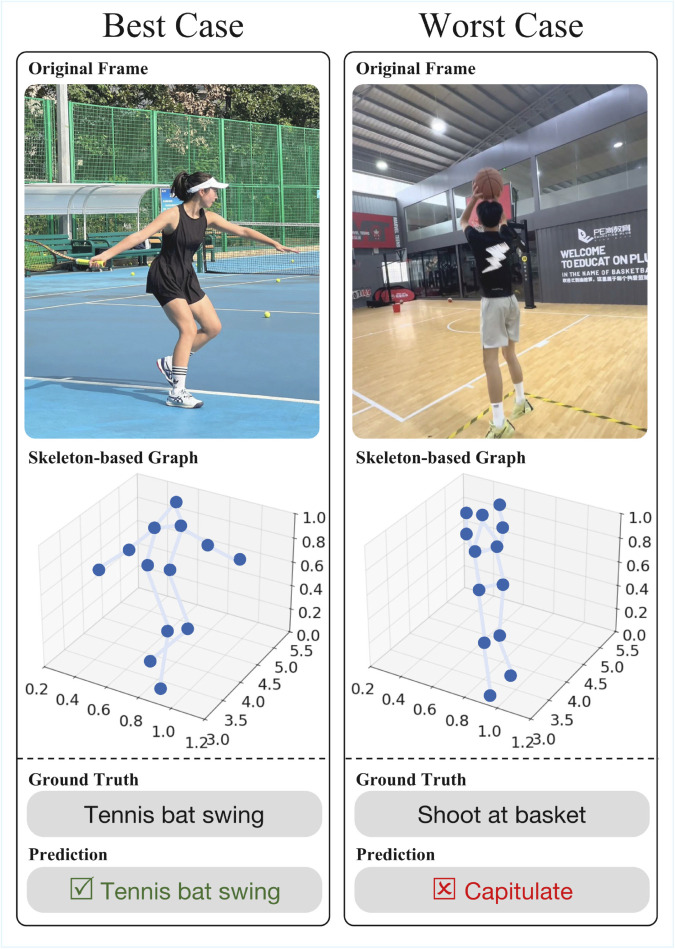
Visualization of SkelFormer’s best and worst prediction cases. The best case (left) demonstrates accurate recognition of ‘tennis bat swing’, with the predicted joints aligning closely with the ground truth. The worst case (right) illustrates a misclassification of the ‘shoot at basket’ as ‘capitulate’, due to the color similarity between the clothing and background, as well as partial occlusion of the upper limbs, which resulted in inaccurate keypoint estimation.

The best and worst prediction cases in Fig 7 highlight both the strengths of SkelFormer and one remaining challenge. In the best case, the model accurately recognizes ‘tennis bat swing’, where the temporal evolution of arm–torso coordination is distinct and the movement phases are clearly separable. In contrast, a misclassification occurs when ‘shoot at basket’ is predicted as ‘capitulate’. This confusion does not arise from errors in the visual domain, since SkelFormer operates purely on skeletal keypoints. Instead, the difficulty stems from the high structural overlap between the two actions in the joint space: both involve similar arm-raising configurations, and the transitional poses can remain nearly identical for many consecutive frames. When such ambiguous segments persist, the temporal patterns available for discrimination become inherently limited, making fine-grained separation challenging even for advanced spatiotemporal models.

A manageable limitation revealed here is that SkelFormer may face difficulty when two actions share long-duration, nearly indistinguishable skeletal postures, reducing the temporal variance that the model relies on. This does not undermine the overall framework but rather reflects a known bottleneck in skeleton-based recognition for gesture pairs with strong structural similarity. A potential future extension is to reinforce the model’s sensitivity to micro-dynamics within these ambiguous intervals. For example, the node diffusion mechanism could incorporate higher-order motion descriptors, such as short-window velocity or acceleration patterns within relay nodes, allowing the model to capture subtle but discriminative timing differences (e.g., the release phase in a shot vs. the static hold in capitulation). Another promising direction is adaptive temporal granularity, where the temporal split module assigns finer temporal resolution precisely to segments with low structural variance. This would allow the SKT Block to focus computational capacity on the most ambiguous frames, improving separability for actions that are nearly indistinguishable at the pose level but differ subtly in motion evolution.

## Conclusion

In this study, we introduce a dynamic hierarchical spatial-temporal graph attention network, SkelFormer, specifically designed for human action recognition. This network dynamically seizes the inherent spatial-temporal topology of the skeleton-based graph, tailored to the specifics of the action. The core of SkelFormer, the SKT Block, with its node concentration and diffusion modules, encapsulates skeleton graph hierarchy information and joint interdependencies. It does so by accumulating representations of proximate nodes on the skeleton-based graph, thereby bolstering the contextual understanding of each distinct action. In terms of performance for skeleton-based action recognition tasks, our model exhibits leading-edge results on well-regarded, public benchmark datasets.
